# Classification of Aggressive Behaviors Based on sEMG Feature Extraction and Machine Learning Algorithm

**DOI:** 10.1093/geroni/igaa057.2262

**Published:** 2020-12-16

**Authors:** Youngjun Kim, Uchechukuwu David, Yeonsik Noh

**Affiliations:** University of Massachusetts Amherst, Amherst, Massachusetts, United States

## Abstract

New surface electromyography (sEMG) feature extraction approach combined with Empirical Mode Decomposition (EMD) and Dispersion Entropy (DisEn) is proposed for classifying aggressive and normal behaviors from sEMG data. In this study, we used the sEMG physical action dataset from the UC Irvine Machine Learning repository. The raw sEMG was decomposed with EMD to obtain a set of Intrinsic Mode Functions (IMF). The IMF, which includes the most discriminant feature for each action, was selected based on the analysis by Hibert Transform (HT) in the time-frequency domain. Next, the DisEn of the selected IMF was calculated as a corresponding feature. Finally, the DisEn value was tested using five different classifiers, such as LDA, Quadratic DA, k-NN, SVM, and Extreme Learning Machine (ELM) for the classification task. Among these ML algorithms, we achieved classification accuracy, sensitivity, and specificity with ELM as 98.44%, 100%, and 96.72%, respectively.

